# Editorial: Recent Advances in Understanding the Pathogenesis of Shiga Toxin-Producing *Shigella* and *Escherichia coli*

**DOI:** 10.3389/fcimb.2020.620703

**Published:** 2020-11-26

**Authors:** Moo-Seung Lee, Jang Won Yoon, Vernon L. Tesh

**Affiliations:** ^1^ Environmental Diseases Research Center, Korea Research Institute of Bioscience and Biotechnology, Daejeon, South Korea; ^2^ Department of Biomolecular Science, KRIBB School of Bioscience, Korea University of Science and Technology (UST), Daejeon, South Korea; ^3^ College of Veterinary Medicine and Institute of Veterinary Science, Kangwon National University, Chuncheon, South Korea; ^4^ Department of Microbial Pathogenesis and Immunology, Texas A&M University, College of Medicine, Bryan, TX, United States

**Keywords:** Shiga toxin-producing *Escherichia coli*, Shiga toxin, hemolytic uremic syndrome, Shiga toxin phage, pathogenesis of STEC

## Introduction

Shiga toxin-producing *Shigella* species and *Escherichia coli* (STEC) are pathogenic bacteria that cause the bloody diarrheal diseases bacillary dysentery (*Shigella* spp.) and hemorrhagic colitis (STEC). Unfortunately, patients with these diarrheal diseases are at risk for developing potentially lethal extraintestinal complications such as hemolytic uremic syndrome (HUS), resulting in acute renal failure, and neurological sequelae such as seizures, cortical blindness, and coma. *Shigella* spp. are among the leading causes of food-borne infectious disease worldwide, especially in children <5 years old, whereas STEC continue to be a public health concern because of the contamination of meat products and vegetables that are widely distributed to consumers. Shiga toxins (Stxs; alternatively referred to as Verotoxins) expressed by the toxin-producing bacteria appear to be primary virulence factors contributing to systemic inflammation and organ dysfunction leading to acute renal failure and neurological impairment. Despite significant progress in our understanding of the genetics, structure, and function of Stxs, the mechanisms of toxin pathogenesis remain to be completely characterized. Recently, numerous studies have suggested that host cellular responses to the toxins (*e.g.*, ER stress, unfolded protein response, cytokine/chemokine production, apoptosis, autophagy) may contribute to pathogenesis. There is an urgent need to improve our understanding of pathogenesis to develop effective therapeutic interventions to prevent or treat Stx-mediated diseases. Articles published in this *Frontiers* Research Topic cover diverse aspects of interactions between host cells, Shiga toxins, and the bacteria that produce the toxins. 

## Recent Updates on *stx2a* Gene Expression as a Virulence Mechanism Contributing to Pathogenesis

Antigenic studies of Stxs and genetic analyses of Stx-encoding bacteriophages have shown that there are two major structural toxin types, designated Shiga toxin type 1 (Stx1) and Shiga toxin type 2 (Stx2). Within each toxin type, there are multiple toxin subtypes. The major toxin subtypes that have been associated with disease in humans are designated Stx1a, Stx2a, Stx2c, and Stx2d. While STEC may express one or more Stx subtypes, epidemiological studies have shown that infection with strains expressing Stx2a are more likely to lead to life-threatening extraintestinal complications. What makes Stx2a-expressing *E. coli* 0157:H7 isolates more pathogenic in comparison to strains expressing Stx1a or Stx1a + Stx2a remains to be fully explored. In this *Frontiers* special topic issue, Hauser et al. demonstrate at a mechanistic level a correlation between elevated induction of toxin expression by *stx*_2a_-encoding phage *in vivo* and lethality in a streptomycin-treated mouse model. The investigators analyzed nine clinical STEC isolates expressing Stx1a + Stx2a, Stx2a + Stx2c, or Stx2a alone. Although there were no major differences in colonization levels in the animals, virulence correlated with the levels of Stx2a expression *in vivo*. The investigators prepared individual deletion mutants of a *stx2a* + *stx2c* strain and showed that, in the presence or absence of ciprofloxacin to induce phage lysis, virulence was primarily associated with Stx2a expression. The correlation of enhanced virulence with Stx2a expression was RecA dependent. Finally, although Stx1a + Stx2a producing strains were relatively avirulent in mice, ciprofloxacin treatment increased lethality in a manner that was neutralized by anti-Stx2a antibodies. Thus, Hauser et al. have made a substantial contribution to our understanding of the paradoxical observation that Stx1a + Stx2a-producing *E. coli* O157:H7 are less likely to cause extraintestinal complications compared to Stx2a-expressing isolates.

## Recent Updates on Potential Reservoirs of STEC

Kim et al. provide a better understanding and comprehensive review with updated information regarding emerging reservoirs of STEC with the potential of transmitting the microorganisms to humans through an environmental cycle. Recently, the utilization of One Health approaches to mitigate zoonotic STEC O157:H7 infections in humans has been emphasized (Garcia et al., 2010). As reviewed by Kim et al. in this issue, there are numerous animal species that have been described as natural reservoirs for this microorganism, including ruminants, monogastrics, birds, fish, amphibians, and invertebrates such as the house fly.

Cattle are well-established reservoirs of STEC, and among these animals, some cattle have been defined as super-shedders as they can shed STEC O157 at higher levels in feces than the others, defined as a level of >10^4^ CFU/g feces (Matthews et al., 2006). In this special topic issue, Teng et al. have extensively characterized a hyper-virulent STEC O157:H7 strain, JEONG-1266, previously isolated from a super-shedder steer (Teng et al., 2016), using comparative genomics and phenotypic analyses. In contrast to many bovine STEC carried asymptomatically in the animals, the JEONG-1266 strain contains a full array of virulence genes characteristic of *E. coli* O157:H7 strains causing disease in humans, including genes encoding Stx2a and Stx2c, type III secretion system (T3SS) structural and effector genes, and genes found in the locus of enterocyte effacement (LEE) region. In addition, the *stx2a*-encoding phage type and the prophage insertion site are identical for JEONG-1266 and *E. coli* O157:H7 strain EC4115, a strain isolated from humans in 2006 during a widespread outbreak of hemorrhagic colitis associated with contaminated spinach. Thus, the findings of Teng et al. support a rationale for One Health approaches in preventing human infections by STEC O157:H7, especially at the farm level, because: (i) JEONG-1266 is genetically close to *E. coli* O157:H7 strain EC4115, a strain known to cause outbreaks in humans, and *E. coli* O157:H7 strain SS17, an isolate from super-shedder cattle; and (ii) all of the above strains are categorized into a clinically important STEC O157 lineage I/II and Clade 8 (Manning et al., 2008; Iyoda et al., 2014). Based on the studies of Teng et al., the identification and culling of super-shedder cattle from the food supply, and the characterization of the STEC strains shed by these animals, will be important to reduce the prevalence of food-borne hemorrhagic colitis and its complications.

## Understanding the Roles of Non-O157 STEC Global Gene Expression and Host Cellular Interactions in Pathogenesis Using an *In Vitro* Intestinal Epithelial Cell (IEC) Infection Model

Only limited information has been available to guide the successful development of efficient preventive measures and therapeutics to human infections by STEC, due in part to the lack of animal infection models and an incomplete understanding of STEC-host interactions. While many studies have characterized pathogenicity using *E. coli* O157:H7 strains, it is important to note that illness caused by non-O157:H7 STEC has continued to increase since 2008. In this *Frontiers* special topic issue, Harrison et al. report their comparative transcriptomic and cytokine expression analyses using an intestinal epithelial cell (IEC) infection model system to understand how *E. coli* core genes and pathotype-specific genes are regulated and how the host cell responds during the transition of the bacteria from the planktonic to the adherent life style. As the authors note, many studies have focused on the transcriptional changes or host cellular responses to single STEC strains. However, the strength of the study by Harrison et al. is that the comparative analyses include two non-O157-STEC strains that cluster in different phylogroups, *E. coli* O26:H11 strain 97-3250 and *E. coli* O145:H28 strain 4865/96, as well as a non-pathogenic, commensal *E. coli* strain. The authors define significant differences in expression of genes involved in amino acid biosynthesis or import and respiration between both STEC strains and the commensal *E. coli.* These data suggest that STEC may utilize diverse strategies to compete with the gut microbiome to successfully colonize. The investigators identified twenty genes that have no homolog with the commensal strain and are differentially regulated in both STEC strains upon adherence to IEC. Three of these genes, located on mobile genetic elements, are candidates for previously unrecognized virulence determinants potentially important for adherence and persistence in the gastrointestinal tract. Overall, the IEC cytokine response to STEC strain 97-3250 appeared to be more robust in comparison to infection with strain 4865/96 or the commensal strain. The authors discuss in more detail how the transcriptomic and cytokine response differences they measure may contribute to pathogenesis, as well as serve as indicators of risk assessment.

## New Insights on Toxin–Toxin Receptor Interactions

Professor Lingwood provides a comprehensive review on recent advances in our understanding of the interactions of Stxs (or Verotoxins) with their primary receptor on mammalian cells, the neutral glycosphingolipid globotriaosylceramide (Gb3). Professor Lingwood reviews the structural requirements for toxin-toxin receptor engagement and the complex intracellular trafficking events, referred to as retrograde transport, which follow toxin binding to Gb3 and subsequent toxin internalization. Finally, the development and testing of toxin receptor analogs as therapeutic agents to prevent or ameliorate toxin-mediated inflammation and pathology is thoroughly and concisely reviewed.

Following STEC adherence, Stxs are released in the intestinal tract. But how the toxins traverse the intestinal epithelial barrier and in what form the toxins circulate to cause systemic inflammation and intoxicate target tissues in the vasculature, kidneys, and central nervous system remain to be clarified. Microvesicles (MVs), ranging in size from 200 to 1,000 nm, are derived from cells by plasma membrane budding and contain cytosolic cargoes. In 2015, Ståhl et al. detected Stx-containing MVs in the renal cortex of HUS patients and in mice orally administered a Stx2a-producing *E. coli* O157:H7 strain (Ståhl et al., 2015). Exosomes are smaller than MVs (30–200 nm) and are formed by the exocytic fusion of multivesicular body membranes with the plasma membrane. Recently, Lee et al. isolated exosomes released from Stx2a-treated human monocyte-derived macrophages to examine the capacity of the cells to produce Stx2a-containing exosomes and to examine the interaction of the exosomes with various Gb3 expressing cells (Lee et al., 2020). Stx2a-containing exosomes were capable of killing Gb3-expressing target cells *via* a mechanism involving caspase-3/7 activation. However, Stx-containing exosomes have not been detected in the circulation in HUS patients. In experiments reported in this special topic issue, Johansson et al. demonstrate that when Stx is delivered to cells within MVs, cytotoxicity of the target cell depends on the endogenous expression of the Stx receptor Gb3. The investigators definitively establish the requirement for target cell Gb3 expression in cytotoxicity by manipulating CHO cells in different ways to add or deplete the toxin receptor. Stx2a delivered to Gb3-expressing target cells enters the retrograde pathway. Interestingly, target cell susceptibility to intoxication requires the endogenous expression of Gb3; the addition of exogenous Gb3-liposomes to Gb3-negative target cells did not confer toxin sensitivity. These data suggest that the target cell must express Gb3 in specific lipid microdomains for retrograde transport and intoxication to occur.

Previously, it was shown that Stxs regulate NLRP3 inflammasome activation to trigger proinflammatory cytokine IL-1β expression *via* a mechanism involving caspase-1 cleavage. This response occurred only in toxin receptor (Gb3) expressing host cells and did not occur in Gb3-negative cells (Lee et al., 2016). In this special topic issue, Wang et al. utilize a series of co-immunoprecipitation studies and mutational analyses to propose that the *Shigella* Type III secretion system effector IpaH4.5, also known as an E3 ubiquitin ligase, promotes inflammasome activation by ubiquitinating and stabilizing the activity of NLRP3, ultimately leading to pyroptosis through caspase-1 cleavage in macrophages infected with the bacteria.

## Conclusions

The contributing articles in this special Research Topic issue of *Frontiers in Cellular and Infection Microbiology* report novel findings regarding recent advances in understanding the pathogenesis of Shiga toxin-producing bacteria.

## Author Contributions

Written and revised by M-SL, JY, and VT. All authors contributed to the article and approved the submitted version.

## Funding

This work was supported by Korea Research Institute of Bioscience and Biotechnology (KRIBB) Research Initiative Program and Basic Science Research Program (2019R1I1A2A01041221) through the National Research Foundation of Korea (NRF), funded by the Ministry of Education. JY was supported by a fund from Research of Korea Centers for Disease Control and Prevention (2017NER54060) and by a research grant of Kangwon National University in 2020.

## Conflict of Interest

The authors declare that the research was conducted in the absence of any commercial or financial relationships that could be construed as a potential conflict of interest.
